# Frequency-Dependent Dynamics of Functional Connectivity Networks During Seizure Termination in Childhood Absence Epilepsy: A Magnetoencephalography Study

**DOI:** 10.3389/fneur.2021.744749

**Published:** 2021-10-25

**Authors:** Jintao Sun, Yihan Li, Ke Zhang, Yulei Sun, Yingfan Wang, Ailiang Miao, Jing Xiang, Xiaoshan Wang

**Affiliations:** ^1^Department of Neurology, The Affiliated Brain Hospital of Nanjing Medical University, Nanjing Medical University, Nanjing, China; ^2^Division of Neurology, MEG Center, Cincinnati Children's Hospital Medical Center, Cincinnati, OH, United States

**Keywords:** childhood absence epilepsy, functional connectivity, magnetoencephalography, seizure termination, multifrequency

## Abstract

**Objective:** Our aim was to investigate the dynamics of functional connectivity (FC) networks during seizure termination in patients with childhood absence epilepsy (CAE) using magnetoencephalography (MEG) and graph theory (GT) analysis.

**Methods:** MEG data were recorded from 22 drug-naïve patients diagnosed with CAE. FC analysis was performed to evaluate the FC networks in seven frequency bands of the MEG data. GT analysis was used to assess the topological properties of FC networks in different frequency bands.

**Results:** The patterns of FC networks involving the frontal cortex were altered significantly during seizure termination compared with those during the ictal period. Changes in the topological parameters of FC networks were observed in specific frequency bands during seizure termination compared with those in the ictal period. In addition, the connectivity strength at 250–500 Hz during the ictal period was negatively correlated with seizure frequency.

**Conclusions:** FC networks associated with the frontal cortex were involved in the termination of absence seizures. The topological properties of FC networks in different frequency bands could be used as new biomarkers to characterize the dynamics of FC networks related to seizure termination.

## Introduction

Childhood absence epilepsy (CAE), with typical electroencephalography (EEG) signals showing 3 Hz spike and wave discharges (SWDs), is one of the most common epilepsy syndromes that occurs in childhood and accounts for 10–17% of epilepsies in children (1, 2). CAE has been historically considered a benign childhood epilepsy syndrome. However, an increasing number of studies have observed that brain function in children with CAE is persistently impaired (3, 4). Repeated seizures and epileptic discharges may impair brain function (5). Therefore, it is necessary to prevent frequent epileptic seizures in children with CAE to alleviate the damage to brain function caused by seizures.

Epileptic seizures were considered the result of hypersynchronous and abnormal discharges among neurons. It was found that the synchronicity of neural activity was enhanced during ictal episodes (6). Some scholars have reported that hypersynchronized neuronal activity increases the chance of epileptic discharges and eventually leads to epileptic seizures (7–9). Therefore, it is possible that the decrease in neuronal synchronization could be a necessary process during seizure termination. However, inconsistent with the previous assumption, the synchronization of neural activity was reported to increase during seizure termination in several studies (10–12). Currently, the specific mechanism underlying seizure termination is still unclear. Understanding the mechanism contributing to seizure termination could offer new insights into its pathophysiological mechanism and be helpful for the development of novel treatments for epilepsy.

The brain can be seen as a complex network in which nodes of a network represent brain areas, and edges reflect either structural or functional connections between different nodes (6). An increasing amount of evidence has indicated that epilepsy is a network disease and that epileptic discharges spread to the whole brain through the network (6, 13–15). Previous studies have also revealed altered functional and structural networks in patients with epilepsy (13, 16–18).

Graph theory (GT) is an ideal tool for quantitative analysis of brain networks. It characterizes topological properties of brain networks through a line of parameters (19, 20). The clustering coefficient and shortest path length are the representative parameters in GT. Also, brain networks could be classified into three types, including small-world networks, random networks, and regular networks, according to these parameters (6, 19). Specific altered topological characteristics were observed in patients with epilepsy reported by several studies (21–23). Moreover, some network parameters were also suggested to be related to clinical features, such as cognitive function and duration of epilepsy, in patients with epilepsy (18, 24). Hence, topological parameters in GT could be used as new biomarkers for describing brain function and identifying the dynamic changes in brain function during seizure termination.

Magnetoencephalography (MEG) is an ideal method for investigating functional networks. MEG can detect magnetic signals from the brain through a non-invasive approach, which is usually used for patient evaluation before epileptic surgery (25, 26). MEG has a higher spatial resolution than EEG, as magnetic signals recorded by MEG are unaffected by the skin and skull (27). Moreover, the temporal resolution of MEG is higher than that of magnetic resonance imaging (fMRI) (27).

The aim of this study was to investigate the dynamic changes in functional connectivity (FC) networks from low- to high-frequency bands during seizure termination in children with CAE by using MEG. We analyzed changes in the pattern and topological properties of the FC networks. In addition, we assessed the association between FC networks and clinical features in children with CAE.

## Methods

### Subjects

Children who were newly diagnosed with CAE were recruited from the Department of Neurology at the Nanjing Brain Hospital and Nanjing Children's Hospital. The inclusion criteria were as follows: (1) typical CAE diagnosed by a neurologist was in line with the International League Against Epilepsy Seizure Classification (2017), (2) bilaterally synchronous 3 Hz SWDs on normal background waves were detected by routine EEG, (3) the patients did not take any medication, and (4) MRI scan results were normal. The exclusion criteria were as follows: (1) history of any diseases or other types of epilepsy and (2) the presence of mental implants, such as pacemakers, which would strongly interfere with MEG recordings. This study was approved by the medical ethics committees of Nanjing Children's Hospital, Nanjing Brain Hospital, and Nanjing Medical University. All subjects and their guardians signed a written informed consent.

### Magnetoencephalography Recording

The MEG data were recorded using a whole-head CTF MEG system with 275 channels (VSM Medical Technology Company, Canada) in a magnetically shielded room at the MEG Center at the Nanjing Brain Hospital. MEG data were acquired at a sample of 6,000 Hz, with noise cancelation of third-order gradients. MEG data recorded in an empty room were used to identify background noise. Before MEG recording, three coils were attached to the nasion and to the left and right preauricular points of each subject to locate the head position of the subject relative to the MEG coordinate system. All metals were also removed from the body of each subject before MEG data acquisition. During MEG recording, all subjects were instructed to stay still with their eyes lightly closed. The head movement of each subject was limited to 5 mm for each data recording. An audio-visual system was used to monitor each subject during MEG recording. At least five continuous data files with a duration of 120 s were collected for each subject. If no SWDs were observed in these data files, another MEG recording was needed, and the subjects were asked to hyperventilate to provoke absence seizures.

### Magnetic Resonance Imaging Scan

All subjects underwent MRI with a 3.0 T scanner (Siemens, Germany). The MRI parameters were as follows: the repetition time was 6,600 ms, the echo time was 93 ms, the field of view was 250 × 250 mm, the flip angle was 9°, and the matrix was 512 × 512. Three markers were placed in the same position used for MEG recording to co-register structural imaging data with the MEG data. All anatomical landmarks digitized during MEG were identifiable in the MRI.

### Data Analysis

**Figure 1:**
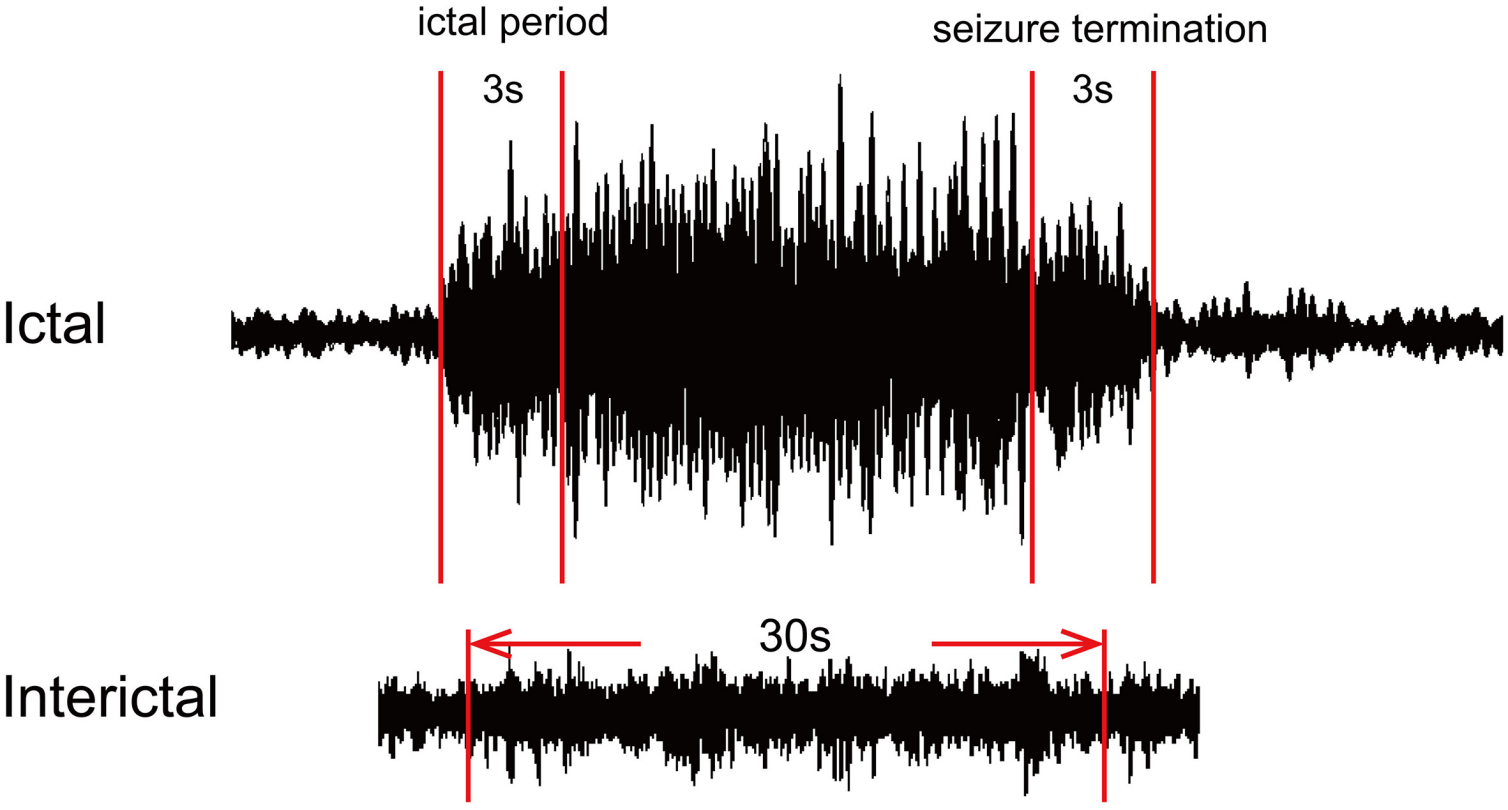
Schematic diagram of magnetoencephalography (MEG) waveform data. For each MEG recording, two segments with a duration of 3 s represented the ictal period and termination period, and one 30-s segment was selected as the interictal period.

All MEG data without any artifacts or background noise were retained. The ictal MEG data were determined using a filter of 1–4 Hz. The SWDs with a duration of more than 4 s were defined as ictal SWDs. The points of seizure onset and offset were identified by two experienced neurologists. Seizure onset was defined by the first spike wave component of SWDs, and seizure termination was defined by the last slow wave in SWDs. We selected three specific segments in each MEG recording for the following analysis. The segment with a duration of 3 s after seizure onset was defined as the ictal period, and the segment with the same duration before seizure offset represented the period of seizure termination. In addition, one segment with a duration of 30 s away from the ictal segment at least 10 s was considered as the interictal period. All selected segments were analyzed in seven frequency bands: delta (1–4 Hz), theta (4–8 Hz), alpha (8–12 Hz), beta (12–30 Hz), gamma (30–80 Hz), ripple (80–250 Hz), and fast ripple (250–500 Hz). Notch filters for 50 Hz and its harmonics were applied to eliminate power-line noise from the MEG data. The details are shown in Figure 1.

### Functional Connectivity Analysis

According to previous studies (28, 29), FC was analyzed at the source level. To construct source neural networks, we used the algorithms to estimate the correlation of each pair of virtual sensors. Specifically, the correlation factors, which were used to analyze the correlation of virtual sensors, were defined as follows:


(1) $$\begin{array}{l} R\left(X_{a},X_{b}\right)=\frac{C\left(X_{a},\;X_{b}\right)}{SX_{a},\;X_{b}} \end{array}$$

In Equation (1), *R* (*X*_*a*_, *X*_*b*_) represents the correlation of a source pair in two locations (“*a*” and “*b*”). *X*_*a*_ and *X*_*b*_ represent the signals from two sources, which were paired for computing connections. *C* (*X*_*a*_, *X*_*b*_) represents the mean of the signals from the two sources. *S*_*xa*_ and *S*_*xb*_ represent the standard deviation of the signals from the two sources. We computed all possible connections for each pair of virtual sensors at the source level to avoid possible bias. If the activity in two source pairs were both increased, the connections were considered as excitatory in MEG processor computations. If increased activity in one source was followed by decreased activity in the other, the connections would be inhibitory. All possible distributions of FC from voxel-based virtual sensors were co-registered to individual participant MRI results (28, 30) and visualized in axial, coronal, and sagittal views. In MRI views, red and blue represent excitatory and inhibitory connections, respectively.

### Graph Theory Analysis

In our study, we used GT to analyze and quantify FC networks at the source level (31). The FC networks in the entire brain consist of nodes and edges, where nodes represent the sources in the brain, and edges indicate the connections between each source pair. Specifically, four measurements including the average strength (*S*), degree (*D*), path length (*L*), and clustering coefficient (*C*) were computed for each possible source pair to quantify the global and local topological properties of FC networks. S indicates the connectivity strength between node pairs. The average *S* reflects the average of all connections in the FC network. *D* refers to the number of links connected to a node, and the average *D* indicates the average degree of all nodes in the FC network (32, 33). *L* indicates the shortest distance between node pairs in the network. The average *L* reflects the tendency of global integration in the FC network (34). *C* represents the likelihood of connection among the neighbors of a node, and the average *C* reflects the tendency of local integration in the FC network (34). The *S, D, L*, and *C* mentioned in our study indicate the average values of the measurements. The details of the equations for GT analysis were described in previous studies (32–34). MEG Processor software was used to analyze the above data (https://sites.google.com/site/braincloudx/). The detailed algorithms of the software were reported in previous articles (28, 29).

To ensure the quality of the data, a threshold was used as a checkpoint. The FC networks were visible in MRI views if the FC values were above the threshold. *t*-values were computed for all source pairs to determine the thresholding of connections. The formula to determine *t*-value was defined as follows:


(2) $$\begin{array}{l} T_{p}=R\sqrt{\frac{K-2}{1-R^{2}}} \end{array}$$

In Equation (2), *T*_*p*_ represents the value of a correlation, *K* represents the number of data points for connections, and *R* indicates the correlation of a source pair. In the present study, we selected the *T*_*p*_ value corresponding to a *p*-value <0.05 as the thresholding to obtain the FC networks and the measurements including *S, D, L*, and *C*.

### Statistical Analysis

Fisher's exact test was used to determine the difference in neural network patterns between different periods in seven frequency bands. Student's *t*-test was used to assess the changes in the network parameters (*S, D, L*, and *C*) between different periods in each frequency band. Partial correlation analysis was utilized to estimate the correlations between clinical characteristics and the network parameters after adjustment for age, sex, and duration of epilepsy. We set the *p*-value threshold as 0.05 in our study. Bonferroni correction was applied for multiple comparisons. Then, we controlled type I errors using the false discovery rate (FDR) controlling procedure. All statistical analyses and computations were performed in SPSS version 20.0 for Windows (SPSS Inc., Chicago, IL, USA).

## Results

### Patients

A total of 22 patients diagnosed with CAE were recruited in the present study. The mean age was 8.23 ± 1.69 years. The gender ratio was 6:16 (male: female). The mean course of epilepsy was 9.73 ± 7.12 months. To minimize the error in the frequency of seizures, we counted the absence seizures observed by parents of children with CAE in the last 1 week to obtain an average of seizure frequency. The average seizure frequency was 8.36 ± 5.41 times/day. A total of 33 ictal MEG data were recorded from all patients. There were 11 patients with one seizure and the other half of patients with two seizures during the acquisition. The duration of the SWDs selected in our analysis is more than 6 s, and there is no overlap between the ictal period and the seizure termination. The details of the clinical characteristics of children with CAE are presented in Table 1.

**Table 1:** Characteristics of the patients with CAE.

**Patient**	**Sex (F/M)**	**Age (years)**	**Duration of disease (months)**	**Frequency of seizures (times/day)**	**Time between diagnosis and the MEG test (day)**
1	M	10	5	6	0
2	F	6	5	2	0
3	F	6	5	2	0
4	F	7	5	10	0
5	M	8	6	10	1
6	F	9	5	10	0
7	M	8	6	7	0
8	F	5	6	2	0
9	F	10	12	5	0
10	F	8	16	5	1
11	F	9	14	5	0
12	F	10	11	6	0
13	F	10	12	8	0
14	F	11	23	8	0
15	F	10	32	8	1
16	F	5	3	8	0
17	F	8	8	8	0
18	M	8	5	20	0
19	F	7	4	20	0
20	F	9	12	4	1
21	M	8	4	15	0
22	M	9	15	18	0

*F indicates female; M indicates male*.

### Network Pattern

At 1–4 Hz, the majority of FC networks (28 of 33 segments) during the ictal period showed strong connections in the parietal cortex and posterior brain regions. No significant difference was observed in the FC network between the ictal period and seizure termination (26 of 33 segments).

At 4–8, 8–12, and 12–30 Hz, the FC networks during the ictal period (30 of 33 segments) were distributed throughout regions in the whole brain, including the frontal cortex, parietal cortex, and posterior brain regions. There was no significant difference in the FC network between the ictal period and seizure termination (32 of segments).

At 30–80 Hz, the FC networks were mainly limited to the frontal cortex (31 of 33 segments) during the ictal period. Notably, the FC networks showed strong connections between anterior and posterior brain regions (29 of 33 segments) during seizure termination compared with the connections between these regions during the ictal period (*p* < 0.05).

At 80–250 and 250–500 Hz, the FC networks showed strong connections in the frontal cortex (31 of 33 segments). No significant difference was seen between the ictal period and the period of seizure termination (30 of 33 segments).

**Figure 2:**
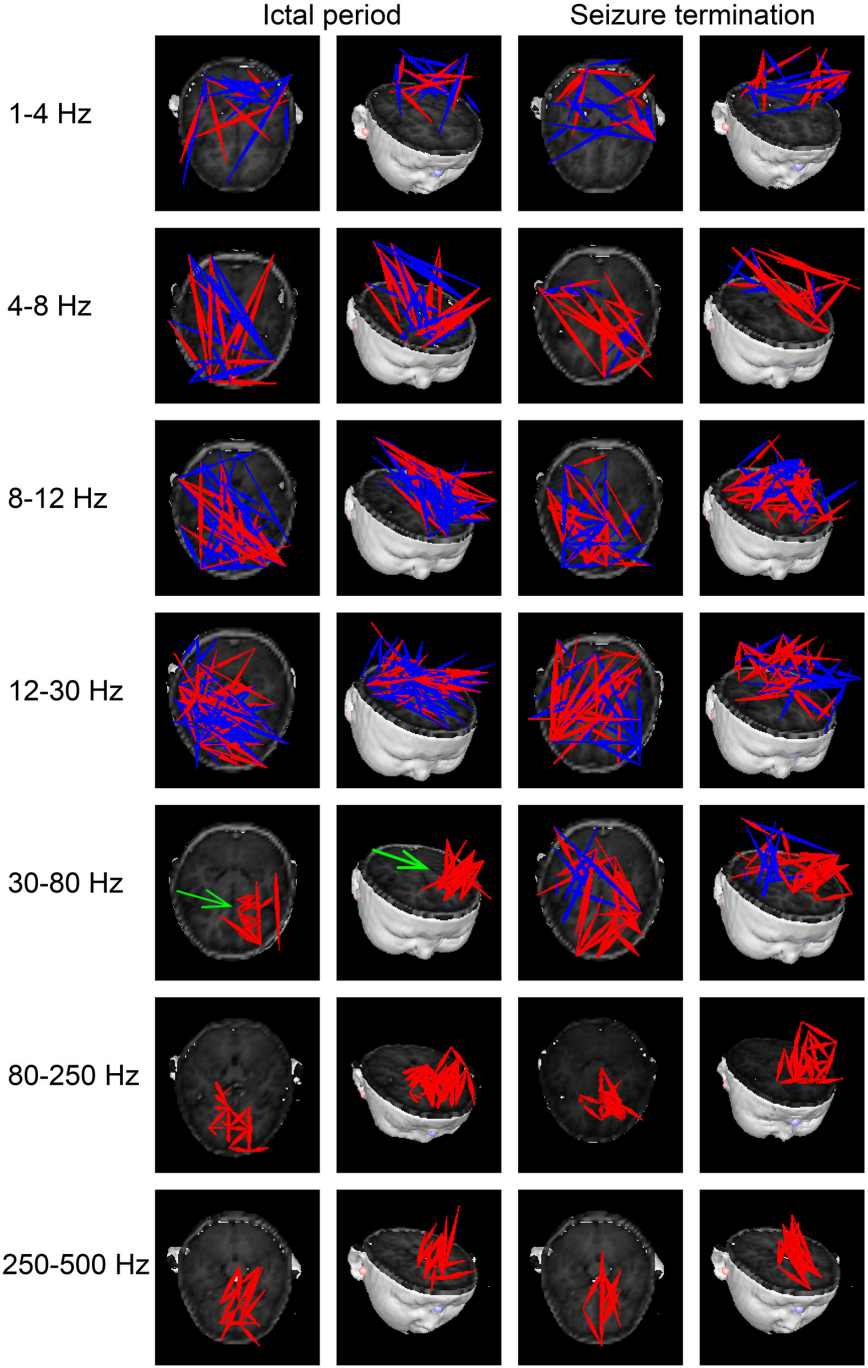
Typical patterns of functional connectivity (FC) networks from childhood absence epilepsy (CAE) patients in seven frequency bands. Red indicates excitatory connections, and blue indicates inhibitory connections. The green arrow indicates significant differences in FC patterns between the two groups. The above results were significant (*p* < 0.05) after false discovery rate (FDR) and Bonferroni correction.

**Figure 3:**
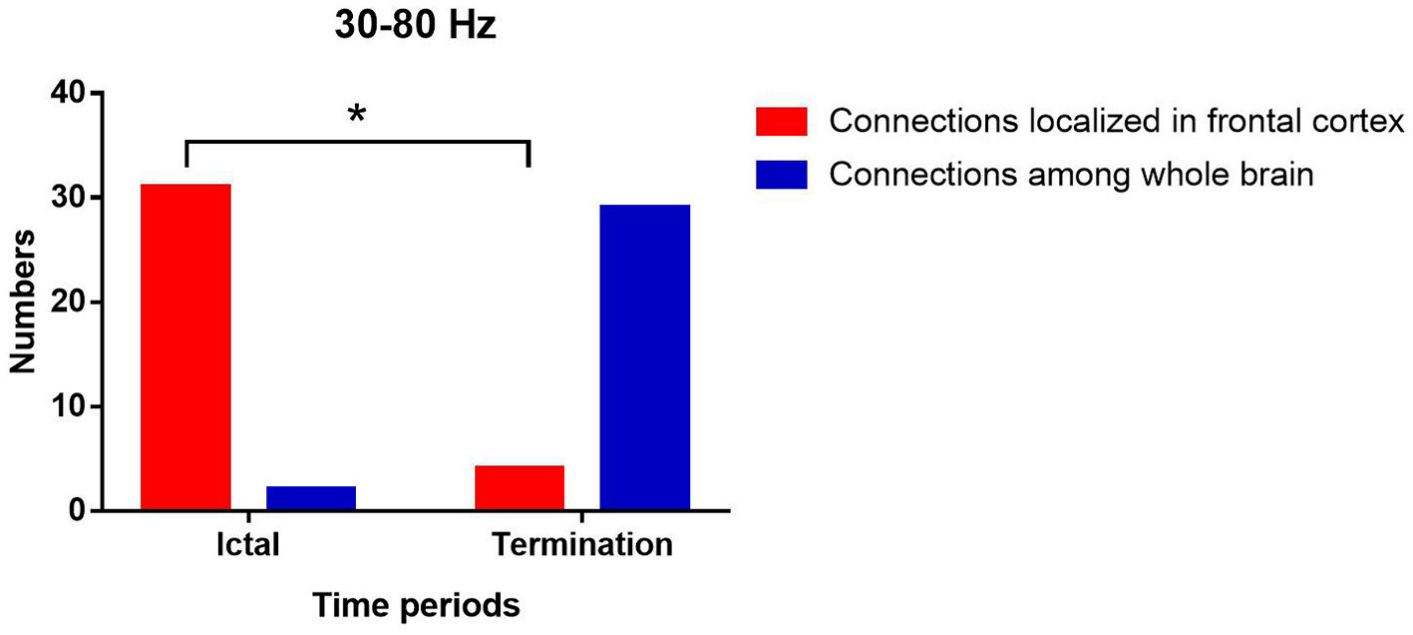
Changes in the number of network patterns localized in frontal cortex in the two periods at 30–80 Hz. P* < 0.05 after FDR and Bonferroni correction.

The above results were corrected by Bonferroni correction and FDR. The details are shown in Figures 2, 3.

### Graph Theory Parameters

**Figure 4:**
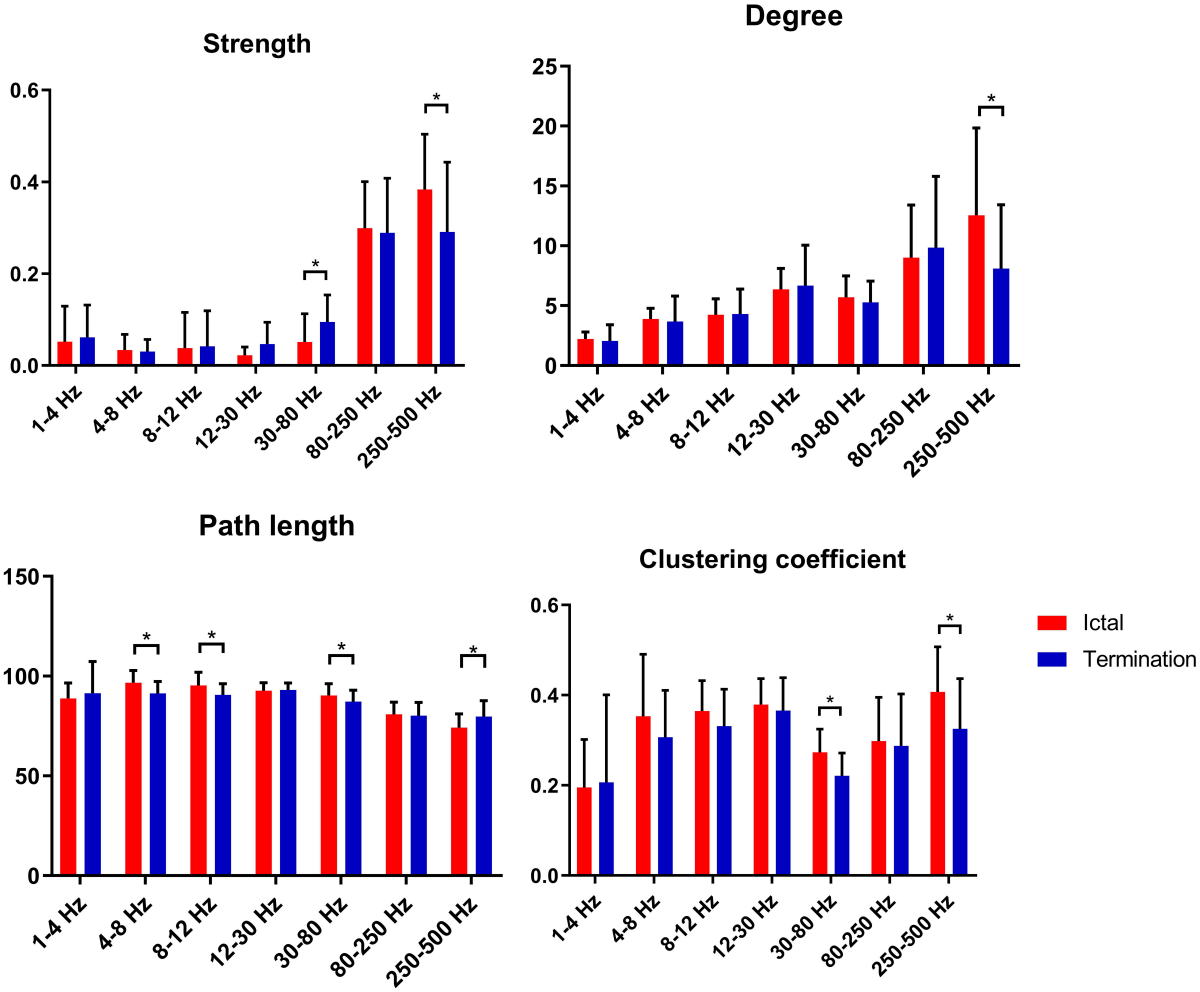
Changes in topological parameters (strength, degree, path length, and clustering coefficient) of FC networks between two groups. P* < 0.05 after FDR and Bonferroni correction.

In our study, we found that S values increased significantly at 30–80 Hz and decreased significantly at 250–500 Hz during seizure termination compared with that during the ictal period (*p* < 0.05). *D* values decreased significantly at 250–500 Hz during seizure termination compared with that during the ictal period (*p* < 0.05). C values decreased significantly at 30–80 and 250–500 Hz during seizure termination compared with that during the ictal period (*p* < 0.05). *L* values decreased significantly at 4–8, 8–12, and 30–80 Hz and increased significantly at 250–500 Hz during seizure termination compared with that during the ictal period (*p* < 0.05). No significant difference was found in other frequency bands. The above results were obtained after Bonferroni and FDR correction. The details are shown in Figure 4.

### Clinical Correlation

**Figure 5:**
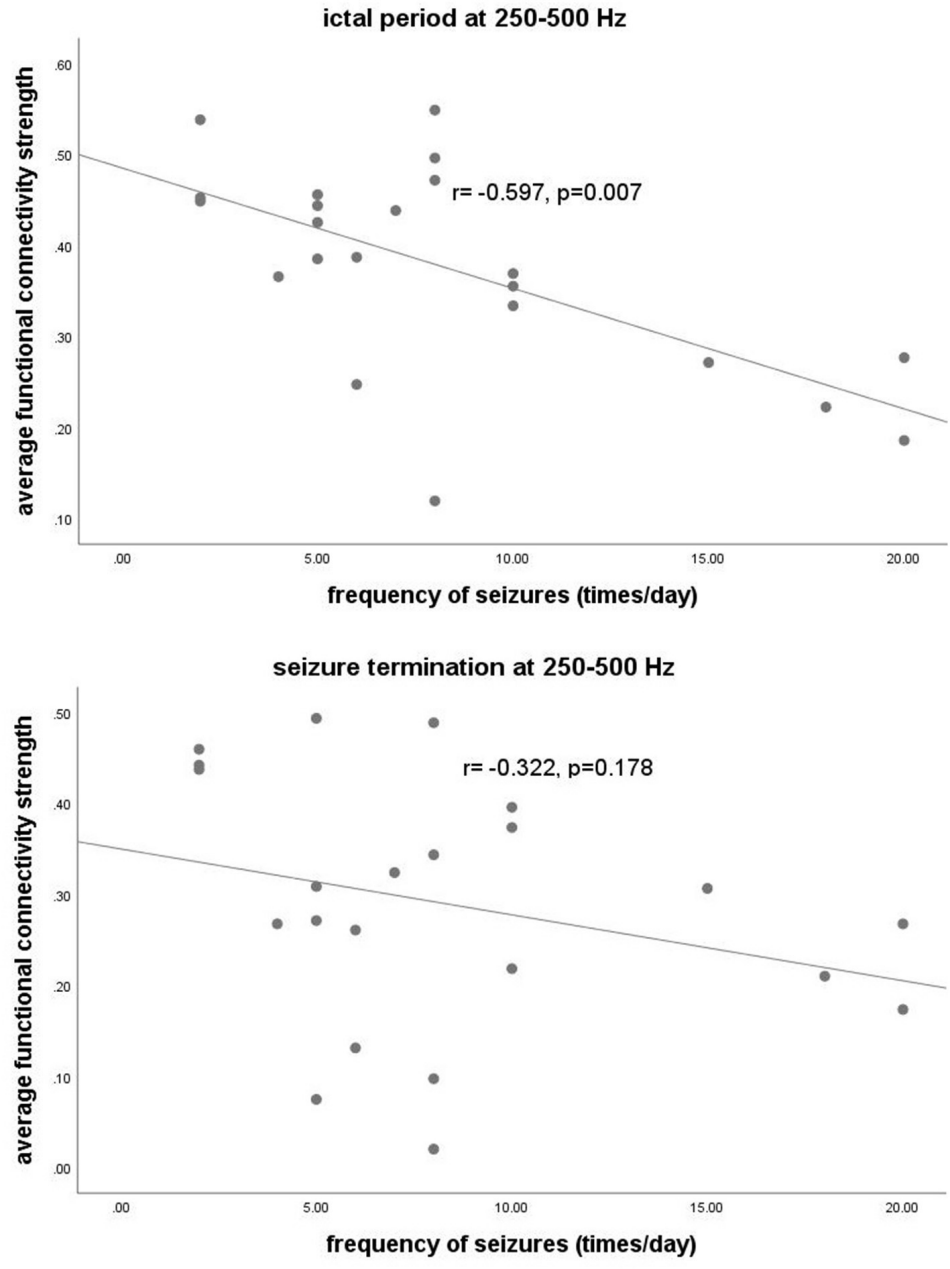
The *y*-axis represents the functional connectivity strength. The *x*-axis represents seizure frequency. Partial correlation analysis showed that the functional connectivity strength at 250–500 Hz during the ictal period was negatively correlated with the frequency of seizures (*r* = −0.597, *p* = 0.007) after adjustment for sex, age, and duration of epilepsy.

At 250–500 Hz, after adjustment for sex, age, and duration of epilepsy, our data revealed that *S* values during the ictal period were negatively correlated with seizure frequency (*r* = −0.597, *p* = 0.007). There was no significant correlation in other frequency bands. The detailed correlation analysis is shown in Figure 5.

## Discussion

In the present study, we investigated the dynamic changes in FC networks during seizure termination in absence seizures using MEG. Our findings revealed that the FC network pattern was changed significantly at 30–80 Hz. Changes in topological parameters of FC networks were also observed in specific frequency bands during seizure termination. In addition, the FC strength at 250–500 Hz was significantly correlated with seizure frequency.

At 30–80 Hz, we found that the FC networks were limited to the frontal region during the ictal period. This finding supported the idea that the frontal cortex is involved in absence seizures. According to previous studies, the frontal cortex has an important role in the initiation and propagation of absence seizures (15, 35, 36). In addition, the localized pattern of FC networks found in our study reflected enhanced connections in local cortical regions and weakened connections among remote brain regions, which was consistent with previous publications (37–39). It is supposed that the increase in short-range synchronization among local neurons along with the decrease in long-range synchronization between distant brain regions might contribute to the generation of epileptic discharges (37–40). Another study also reported that synchronization in local neural populations plays a critical role in initiating seizures (41). Hypersynchronized FC networks involving the frontal cortex were a critical factor in the onset of absence seizures (15). Recent publications, which suggested that epileptic discharges during absence seizures originate from the frontal lobe, could also partially explain the enhanced frontal FC networks observed in the present study during the ictal period in absence seizures (36, 42). During seizure termination, we noticed obvious connections between posterior and anterior brain regions replacing the limited network connections localized in frontal regions at 30–80 Hz. This difference in connections suggested that the long-range synchronization among remote brain regions was increased, leading to an altered FC network pattern among brain regions, including the frontal lobe, during seizure termination. In several studies, ictal epileptic networks were fragmented at seizure onset and then merged with other cortical regions gradually by long-range synchronization (38, 43, 44). Another study also found that the cortical regions involved in epileptic networks during seizure termination were wider than those at seizure onset, indicating that the changes in the patterns of FC networks participated in seizure termination (45). Moreover, altered function in the frontal lobe during seizure termination was reported by recent investigations (46, 47). Given the literature mentioned above as well as the results found in our study, we speculated that the changes in FC network patterns associated with the frontal cortex were involved in the termination of absence seizures. Further investigations exploring the specific mechanism of seizure termination involving the frontal cortex are needed in the future.

At the same time, we noticed frequency-dependent patterns of FC networks from low- to high-frequency ranges during absence seizures in our study. A previous study on absence seizures demonstrated that during the ictal period, FC networks were more likely to localize in posterior brain regions at low-frequency ranges and localize in anterior regions at high-frequency bands, which was consistent with our results (48). Other studies also found distinct alterations of networks in different frequency bands in CAE patients (13, 30, 47, 49, 50). According to a previous report, the types of connections in different frequency bands had specific interactions that allowed information to be integrated or shared at different spatiotemporal levels (48). For instance, low-frequency neural interactions were used to integrate information over a wider range of cortical regions, whereas high-frequency neural interactions were suited to communicate with neighboring neurons (30, 51, 52). Therefore, the changes in the patterns of FC networks observed in specific frequency bands instead of all frequency bands during seizure termination could be partially explained by the different roles that neural activity played in different frequency bands. These changes and their implications are worthy of further research.

### Graph Theory Parameters

GT analysis in our study revealed increased S values and decreased *C* and *L* values at 30–80 Hz as well as decreased S, decreased D, decreased C, and increased *L* values at 250–500 Hz. These findings suggested that the topological properties of FC networks in patients with CAE were altered during seizure termination compared with those during the ictal period.

At 30–80 Hz, *S* values increased during seizure termination compared with those observed during the ictal period, suggesting that the synchronization of FC networks in the whole brain was enhanced significantly. One study on temporal lobe epilepsy also found similar enhanced synchronization before termination in the gamma frequency band, which was consistent with our results (53). Furthermore, increased synchronization of neural activity was also observed before seizure offset in other studies and was thought to be related to seizure termination (10, 11, 44, 54, 55). Another study reported that vagal nerve stimulation (VNS), a non-invasive treatment for refractory epilepsy, may influence cortical activity by increasing gamma frequency synchrony, reducing the likelihood of seizure onset, or promoting seizure termination (41). In addition, we noticed that *C* and *L* values, which were considered as representative parameters of small-world networks, were decreased significantly during termination compared with those during the ictal period, indicating that the topological characteristics of FC networks were altered toward random networks. As reported by several studies, epileptic networks were more likely to be random networks characterized by decreased *C* and *L* values during the interictal period and were likely to function as regular networks accompanied by increased *C* and *L* values during the ictal period (13, 34, 56). Moreover, it was reported by other scholars that the *C* and *L* topological properties of epileptic networks increased at seizure onset and then decreased before seizure offset, again suggesting randomization trends in epileptic networks during seizure termination (38, 57, 58). Given that randomized networks are considered as the usual topological pattern of epileptic networks in the interictal period, we suppose that the topological properties of FC networks observed during termination might serve as transitional states from ictal to interictal periods, thus, combining the double topological characteristics in ictal and interictal periods.

At 250–500 Hz, the present study revealed that *S, D*, and *C* values were decreased, and *L* values were increased during the period of termination compared with those during the ictal period, which indicated that the topological organization during seizure termination was far from the optimal structure for information propagation. High-frequency oscillations (HFOs), especially fast ripples (ranging from 250 to 500 Hz), were reported to be related to the generation of seizures in recent literature (22, 30, 49). Therefore, we speculate that the topological organization at 250–500 Hz during the termination phase was a less efficient structure that might not be helpful for continuous epileptic discharges from the seizure onset zone (SOZ) and might promote seizure termination. The generation mechanisms of HFOs and gamma oscillations (30–80 Hz) were not the same, and these two oscillations have distinct patterns of network dynamics during the seizure period (59–61). Therefore, the differences in generation mechanisms could partially explain the different network patterns and topological properties we found at 30–80 and 250–500 Hz during seizure termination.

In general, we propose that the changes in parameters of network topology observed during termination at different frequency bands could be used as new biomarkers for characterizing the subtle dynamics of networks of CAE during termination, although the specific causal correlations between topological parameters and seizure termination warrant further investigation.

### Clinical Correlation

In the present study, we found significant correlations between the *S* values of topological parameters at 250–500 Hz during the ictal period and the seizure frequency of CAE patients. According to previous studies, the brain region generating HFOs represents the SOZ and is involved in the initiation and propagation of epilepsy (22, 61). In recent years, HFOs have been used for mapping epileptic foci for epilepsy surgery (62, 63). Furthermore, several studies have found that HFOs are associated with the severity of seizures (43, 64–66). In our study, we suppose that the topological parameters of networks at 250–500 Hz could be considered as new biomarker options to estimate the severity of absence seizures.

### Limitations

However, there are several limitations in this study. First, the sample size of this study was relatively small, which may have an impact on the results. Further studies investigating the potential mechanisms underlying seizure termination from the perspective of FC networks should be performed in a larger cohort of patients. Second, we did not discuss the potential effect of deep brain areas (DBAs), such as the thalamus, on seizure termination since the spatial resolution of MEG for deep brain detection is still under debate. In the future, this issue could be resolved by taking measurements with a moving MEG through a wearable system. Such a device could improve the spatial resolution of MEG in DBAs and acquire more accurate results than routine MEG devices (67). Third, although we minimized the artifacts, artifacts from electromyography and other signals may still be included in the MEG recordings and affect our results. Further investigations should be performed to determine whether artifacts have been eliminated completely. In addition, given the limitations of using single software program in our study, other imaging software is needed to confirm the repeatability of the results in further studies. Last but not least, although functional network analysis is one method to study the mechanism of seizure termination, the results and conclusion in the present study need to be verified by further investigations using different methods.

## Conclusion

In conclusion, our study demonstrated that the FC networks during seizure termination differed from those during the ictal period in specific frequency bands. The pattern of FC networks involving the frontal cortex was altered during seizure termination, suggesting that the frontal lobe possibly plays a critical role in seizure termination. The topological parameters of FC networks changed during seizure termination, provided new biomarkers that could be used to characterize the dynamics of seizure termination, and were helpful for further investigating the specific relationships between topological properties of the network and seizure termination. The *S* values of topological parameters at 250–500 Hz were found to be correlated with seizure frequency, offering a new biomarker that could be used to estimate the severity of absence seizures.

## Data Availability Statement

The raw data supporting the conclusions of this article will be made available by the authors, without undue reservation.

## Ethics Statement

This study was approved by the Medical Ethics Committees of Nanjing Children's Hospital, Nanjing Brain Hospital, and Nanjing Medical University. Written informed consent to participate in this study was provided by the participants' legal guardian/next of kin.

## Author Contributions

JS, YL, KZ, and XW designed the research. JS, YS, AM, YW, and JX analyzed the data. JS, KZ, YS, and YW recruited the participants and acquired the images. JS wrote the manuscript. XW revised the manuscript. All authors approved the final submitted version and agreed to be accountable for its content.

## Funding

This study was supported by the General Program of Natural Science Foundation of Jiangsu Province (Grant No. BK20191127), the Health Department of Jiangsu Province (Grant No. H2018062), the Medical and Health International Cooperation Project of Nanjing Municipal Science and Technology Bureau (Grant No. 201911044), and the National Natural Science Foundation of China (Grant No. 82071455).

## Conflict of Interest

The authors declare that the research was conducted in the absence of any commercial or financial relationships that could be construed as a potential conflict of interest.

## Publisher's Note

All claims expressed in this article are solely those of the authors and do not necessarily represent those of their affiliated organizations, or those of the publisher, the editors and the reviewers. Any product that may be evaluated in this article, or claim that may be made by its manufacturer, is not guaranteed or endorsed by the publisher.
